# Rabies and Canine Distemper Virus Epidemics in the Red Fox Population of Northern Italy (2006–2010)

**DOI:** 10.1371/journal.pone.0061588

**Published:** 2013-04-22

**Authors:** Pierre Nouvellet, Christl A. Donnelly, Marco De Nardi, Chris J. Rhodes, Paola De Benedictis, Carlo Citterio, Federica Obber, Monica Lorenzetto, Manuela Dalla Pozza, Simon Cauchemez, Giovanni Cattoli

**Affiliations:** 1 Medical Research Council Centre for Outbreak Analysis and Modelling, Department of Infectious Disease Epidemiology, Imperial College London, London, United Kingdom; 2 Food and Agriculture Organization Reference Centre for Rabies and World Organisation for Animal Health Collaborating Centre for Diseases at the Human-Animal Interface, Research and Development Department, Istituto Zooprofilattico Sperimentale delle Venezie, Legnaro, Padova, Italy; 3 Regional Centre for Veterinary Epidemiology, Istituto Zooprofilattico Sperimentale delle Venezie, Legnaro, Padova, Italy; 4 Istituto Zooprofilattico Sperimentale delle Venezie, Sezione territoriale di Belluno, Belluno, Italy; Institute for Animal Health, United Kingdom

## Abstract

Since 2006 the red fox (*Vulpes vulpes*) population in north-eastern Italy has experienced an epidemic of canine distemper virus (CDV). Additionally, in 2008, after a thirteen-year absence from Italy, fox rabies was re-introduced in the Udine province at the national border with Slovenia. Disease intervention strategies are being developed and implemented to control rabies in this area and minimise risk to human health. Here we present empirical data and the epidemiological picture relating to these epidemics in the period 2006–2010. Of important significance for epidemiological studies of wild animals, basic mathematical models are developed to exploit information collected from the surveillance program on dead and/or living animals in order to assess the incidence of infection. These models are also used to estimate the rate of transmission of both diseases and the rate of vaccination, while correcting for a bias in early collection of CDV samples. We found that the rate of rabies transmission was roughly twice that of CDV, with an estimated effective contact between infected and susceptible fox leading to a new infection occurring once every 3 days for rabies, and once a week for CDV. We also inferred that during the early stage of the CDV epidemic, a bias in the monitoring protocol resulted in a positive sample being almost 10 times more likely to be collected than a negative sample. We estimated the rate of intake of oral vaccine at 0.006 per day, allowing us to estimate that roughly 68% of the foxes would be immunised. This was confirmed by field observations. Finally we discuss the implications for the eco-epidemiological dynamics of both epidemics in relation to control measures.

## Introduction

**Figure 1:**
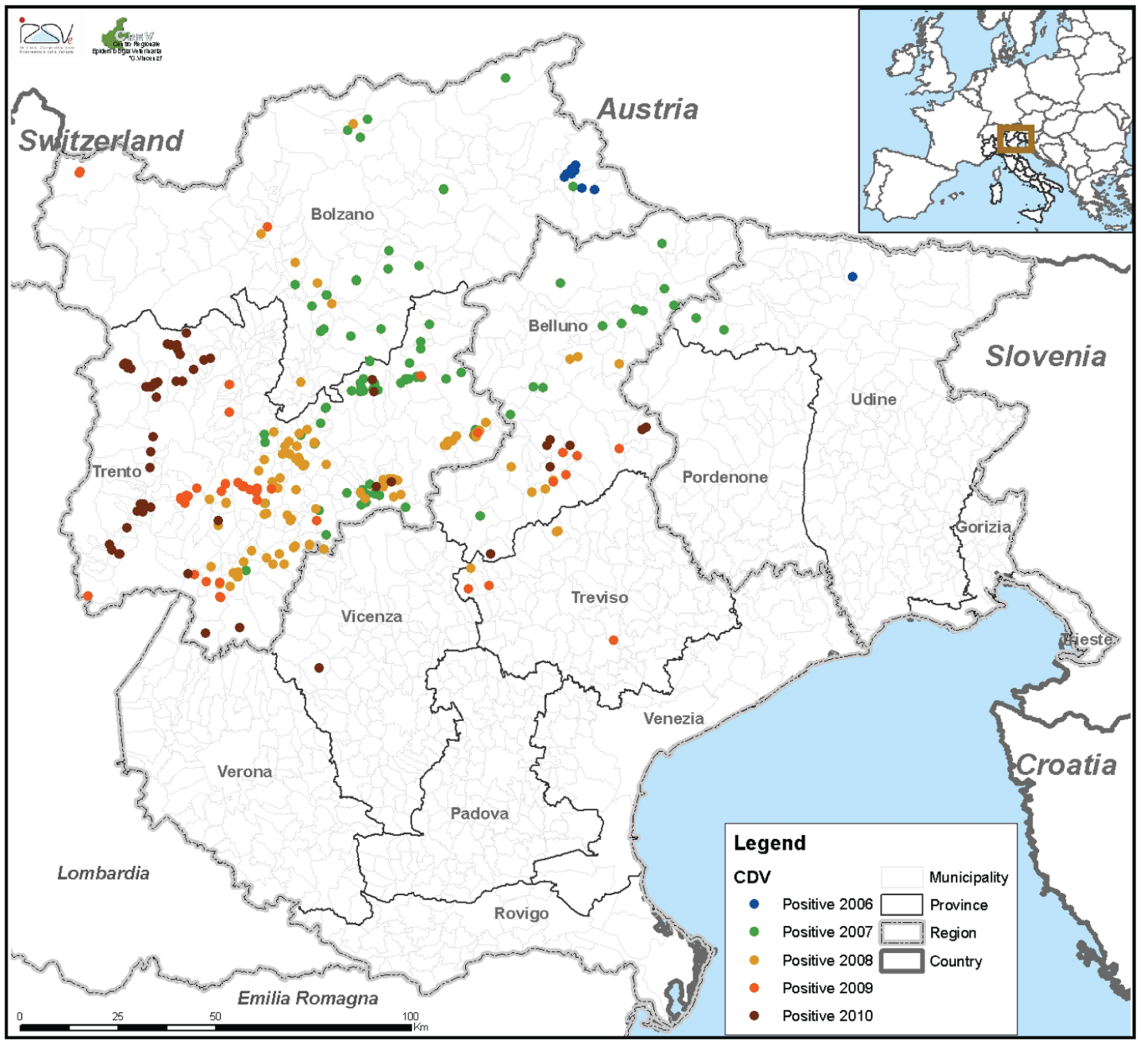
Spread of CDV in wildlife in north-eastern Italy between 2006 and 2010. Cases in Lombardia are not shown. Colour coding indicates the year of observations, with the epidemics starting in the Bolzano and Udine province in 2006 and having spread to Trento, Vicenza and Belluno province by 2010. Most cases of CDV concentrated in the Trento province.

Since 2006 the red fox (*Vulpes vulpes*) population of the Alpine regions of north and north-eastern Italy has been subjected to a persistent epidemic of canine distemper virus (CDV). The outbreak has affected widely different regions of northern Italy [1], [2] which share international borders with Switzerland and Austria to the north and Slovenia to the east (Figure 1).

**Figure 2:**
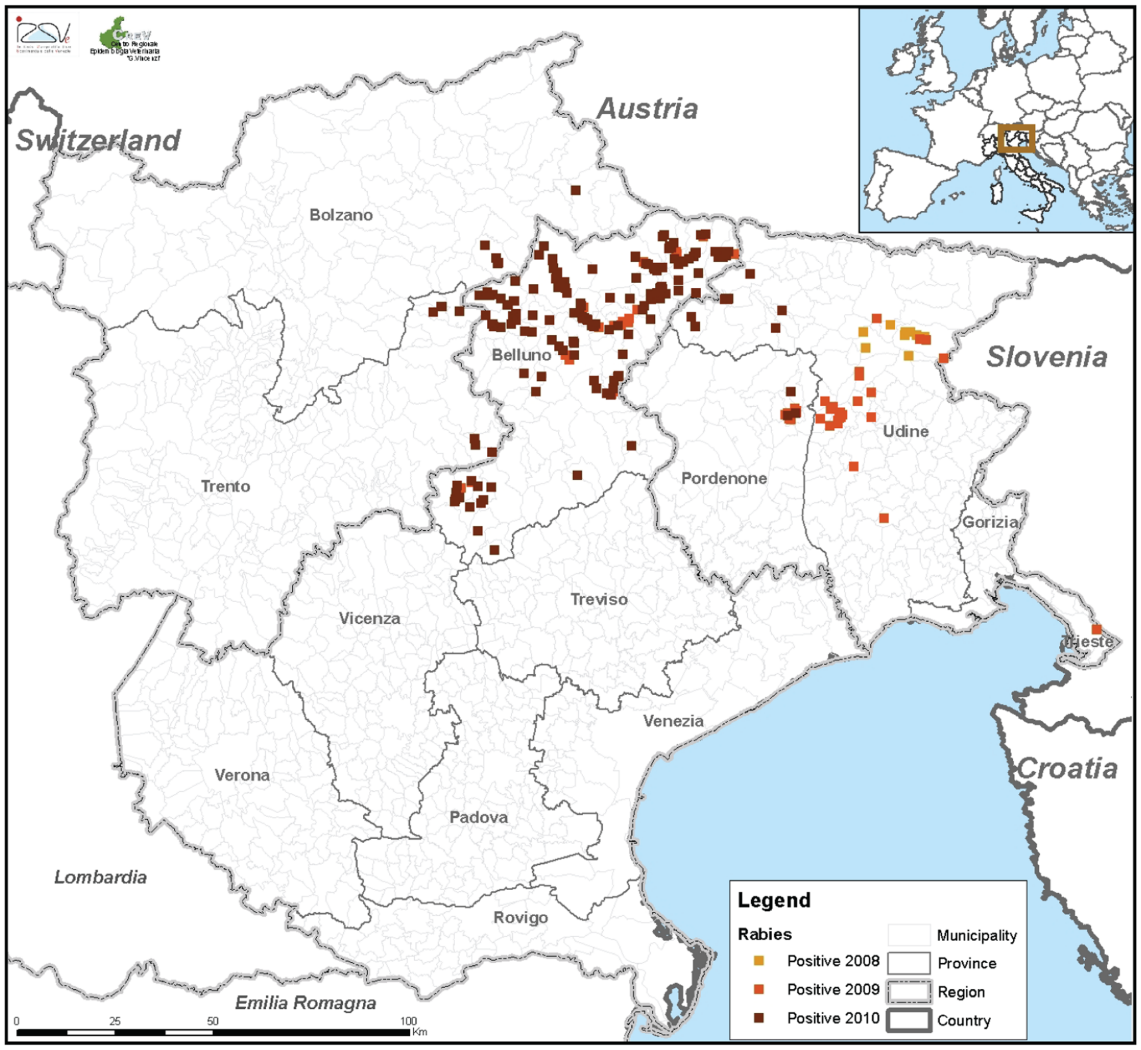
Spread of rabies in wildlife carnivores between 2008 and 2010. Again colour coding indicates the year of observations, with the epidemics starting in the Udine province in 2008 and most detected cases observed in the Belluno province during 2010.

Of more significant concern from a public health point of view, however, was the notification in 2008 of confirmed cases of rabies in wild red foxes in the province of Udine (in the Friuli Venezia Giulia region) close to the border with Slovenia (Figure 2). In Italy, the risk of rabies re-introduction from the bordering areas has long been recognised. The north-eastern territories were affected by rabies in the 1970s and 1980s, and more recently from 1991 to 1995, linked [3] to infections in Austria and the nearby territories of former Yugoslavia (now Slovenia). Vaccination campaigns using oral rabies vaccine have been conducted to target the red fox population in these areas in 1989 and between 1992 and 2004 [3], [5]. Prior to 2008, the last case of rabies was diagnosed in a fox on the border with Slovenia in December 1995, and Italy had been classified as rabies-free since 1997.

The new epidemiological evidence prompted the introduction of a programme of oral vaccination of foxes, mandatory vaccination of domestic dogs and heightened disease surveillance in affected areas by veterinary authorities. At the same time officials initiated regional public health campaigns to increase awareness of both public and animal health risks.

CDV (genus *Morbillivirus*, family Paramyxoviridae) has a wide host-range [5] and evidence of infection has been confirmed in mammalian species belonging to three different orders, *Carnivora*, *Artiodactyla* and *Primates* [6]–[8]. CDV is nevertheless particularly common in *Canidae* species such as dogs (*Canis lupus familiaris*) and foxes (*Vulpes vulpes*) and widespread with outbreaks reported in both domestic and wild carnivores in many European countries [9]–[15].

The rabies virus (genus *Lyssavirus*, family Rhabdoviridae) is able to infect all mammalian species, though susceptibility varies among species [16]. Rabies is a fatal zoonosis and its occurrence is of serious concern to public health authorities. Fox rabies has disappeared in Western Europe [17], [18] and to date nineteen countries, namely Austria, Belgium, Cyprus, Czech Republic, Denmark, Finland, France, Germany, Greece, Iceland, Luxembourg, Norway, Portugal, Spain, Sweden, Switzerland, Lichtenstein, the Netherlands and the United Kingdom have been declared free of wildlife rabies, with sporadic imported cases in domestic animals and humans [19]–[21]. Norway has recently experienced a case in an arctic fox (*Vulpes lagopus*) in the Svalbard archipelago [22].

Because of the evident risk to humans there has been a long-standing interest in the development and refinement of mathematical models for rabies virus infection in wildlife reservoirs [23]–[27]. Such models helped in understanding observed patterns of disease dynamics and spread, and permitted the assessment of mitigation strategies [28]. By contrast, the epidemiology of CDV in red foxes has been much less studied, probably because CDV is not a threat to human health, and much remains to be learned.

In the context of emerging and zoonotic diseases, the importance of wildlife monitoring and surveillance has long been recognised [29]–[31], and is becoming critical as the threat of emerging disease is increasing [31]–[34] at least partly due to growing interactions between wildlife and humans. Two monitoring strategies can be broadly defined as passive and active surveillance [35]–[37]. While passive surveillance in wildlife studies relies on opportunistic observations or collection/sampling of diseased or dead animals by either untrained (i.e. member of the public) or trained personnel (i.e. rangers, hunters), active surveillance may include systematic trapping and/or outbreak investigation by specially trained personnel (i.e. veterinary services, biologists). The two strategies can report either on living animals or animals found dead or both. Translating prevalence information based on dead individuals, or sero-prevalence depending on diagnostic method, into information on incidence of disease is non-trivial. For instance, considering a hypothetical extreme example of a rabies epidemic where surveillance is based solely on specimens collected on dead animals, the proportion of positive samples may largely overestimate the incidence of rabies in the population because infected animals, dying at a much higher rate due to virulence, are much more likely to be sampled than others. It is therefore important to develop statistical methods that can correct for this surveillance bias, and allow reliable estimation of incidence at the population level using data collected from the different surveillance streams. Thus in combination with the implementation of surveillance programs, the development of methodologies to appropriately analyse their results is vital.

Once incidence has been described, a good understanding of the transmission dynamics of the disease is important to properly plan, implement and monitor the impact of control strategies. For instance, the rate at which disease transmission occurs must be known to determine the vaccine coverage needed to eliminate a disease [23]. For many diseases, including CDV, such information is lacking and for many others, including rabies, the use of estimates based on a distinct species or geographical location has been criticised [36], [38].

This study aims to develop an initial understanding of the epidemiological dynamics of rabies and CDV transmission in the red fox population of north-eastern Italy. In what follows we first describe the epidemiological picture of the ongoing outbreak of CDV and the case pattern of rabies infection in this wildlife population in the period 2006–2010. We then develop a method, based on basic compartmental mathematical models and likelihood estimation, to 1) derive estimates of incidence corrected for the surveillance bias that may occur when data are collected on both living and dead animals and 2) estimate the parameters of transmission of both pathogens. While prior to the end of 2009, monitoring was part of passive surveillance, after this date, when the first rabid cases in Belluno province were detected, the monitoring of both diseases became more active and systematic. Before the end of 2009, it is possible that the monitoring procedure was biased with CDV positive foxes being preferentially reported. Here we estimated the magnitude of this bias. Finally we estimated the rate of rabies vaccination intake following the emergency oral vaccination campaign to understand and assess the impact of the vaccination program. Rabies and distemper share the same host in the affected geographic regions and have some similar epidemiological characteristics. In an attempt to investigate the extent to which these 2 viral diseases may influence each other's dynamics we also developed and tested a prototype co-circulation model (Text S1).

## Materials and Methods

This study includes rabies and CDV epidemiological data gathered in the period 2006–2010. Preliminary results suggest an increase in the number of cases of CDV in foxes in 2011 and 2012 [39]. The most recent case of rabies in foxes was detected in February 2011. In this section we present the method of data collection, mathematical models describing the dynamics of the two diseases, and how they can be used to gain insight in this particular setting.

### Samples collection and emergency oral rabies vaccination campaign

Samples were collected initially under the framework of a specific research programme aimed at investigating the phylogenetic characteristics of the CDV circulating in north-eastern Italy [1] and, since 2008, under the passive national surveillance strategy for rabies. A large proportion of animals from which samples were taken, were found dead by hunters and forest rangers, collected and submitted to the Istituto Zooprofilattico Sperimentale delle Venezie (IZSVe) for analysis. In a few cases animals were killed due to the observation of neurological signs. Through awareness and sensitization programs the local population has been encouraged to report the presence of dead animals or of animal showing atypical behaviors. CDV infection was confirmed by laboratory diagnosis with Reverse Transcriptase-Polymerase Chain Reaction (RT-PCR) and direct-immuno-fluorescence assay on brain and lung tissue [1]. Rabies infection was diagnosed with direct-immuno-fluorescence assay on brain, typed by RT-PCR and sequencing [4], [40].

Italian authorities in co-ordination with Slovenia and Austria have adopted measures to control the epidemic and protect public health including compulsory rabies vaccination of dogs and domestic herbivores at risk of infection, prohibition of hunting with dogs, an obligation to keep dogs on a leash, enhanced passive surveillance in wildlife and implementation of targeted oral fox vaccination [41]. In north-eastern Italy, several oral fox vaccination campaigns using aerial distribution of rabies vaccines baits have been implemented since 2009. In January 2010 a month-long emergency oral vaccination campaign was initiated using SAD B19 vaccine baits. As vaccine was distributed only below the freezing point altitude, the suitable distribution was relatively small (8000 km^2^). A much wider campaign (suitable area: 28000 km^2^) followed from April to June 2010 using SAG2 vaccine baits. During August-September 2010 and November-December 2010 follow up campaigns had similar coverage (see [42], [43] for detailed information including coverage maps). Oral fox vaccination programs are currently still being implemented. To monitor the impact of the 2009–2010 campaigns on fox populations, foxes were sampled following vaccination and were tested through fluorescent antibody virus neutralization test: a fox was considered protected if the test detected an antibody titre ≥0.5 IU/ml [40], [44]. For this reason, the number of culled foxes and consequently the total number of samples submitted to the laboratory for analysis increased considerably since late 2009 and throughout 2010.

### Modelling the dynamics of CDV and rabies epidemics

#### Red fox ecology in Italy

Information on the spatial distribution of the fox population in northern Italy is limited. Despite a few cases of distemper detected in urbanized areas, the places where both diseases have been mainly reported are rural farm land and forested areas in the hilly and mountainous Pre-Alpine and Alpine regions. Given prediction of fox density according to habitat type [28], data on the fox population density in the South Western area of Belluno [45], in Friuli Venezia Giulia and in Piacenza province [46] and lowland Italy [47], we assumed that disease-free density of red foxes is approximately 1 fox/km^2^. The lack of ecological data related to the host population in the area is considerable, so the seasonality in fox birth, or the dispersal of juveniles in specific periods of the year, was not included in our model.

In the absence of virus, we assume that the fox population is governed by a logistic growth to some carrying capacity *K* = 1 foxes/km^2^. Healthy foxes have a mean natural life-span of 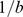
days with a *per capita* birth rate of *a* foxes per day (see Table 1 for specific values and references). The population has a growth rate, 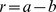.

**Table 1:** Parameters for the rabies and CDV epidemic models in foxes.

parameter	symbol	Assumed values [reference]
Ecology		
Fox carrying capacity	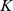	1/km^2^ [28], [45]–[47]
*per capita* birth rate	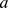	1/365 days [23]
*per capita* death rate	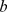	1/730 days [23]
Rabies		
mean viral incubation period	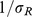	28 days [23]
mean duration of infection	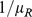	5 days [23]
transmission parameter	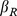	Estimated
Vaccination rate	*v*	Estimated
CDV		
mean viral incubation period	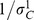	10 days [5]–[7], [48]
proportion of foxes that die from CDV	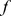	0.5 [5], [50]
mean duration of infection (for foxes that will recover)	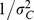	10 days [7], [48]
mean duration of infection (for foxes that will not recover)	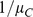	10 days [7], [48],
transmission parameter	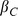	estimated
Bias in preferentially reporting/collecting CDV positive animals prior to the last quarter of 2009	*z*	estimated

Using a maximum likelihood approach, we estimated transmission, vaccination and bias parameters, given values of the other parameters drawn from the literature. These parameters clearly point at a higher virulence of rabies' virus both in term of case fatality and life expectancy once infectious.

#### Disease transmission and within-host considerations

The transmission routes between wildlife hosts differ significantly for CDV and rabies virus, but the natural course of the diseases within carnivores has similarities.

Transmission of rabies between foxes is by biting of an uninfected fox by an infected one [23], [24]. Rabies virus is concentrated in saliva and the bite wound facilitates entry of virus into muscle tissue from where it migrates to the nervous system. Therefore, close contact between susceptible and infected foxes is required for transmission. Because virus migrates from the tissue-wound point of entry to the central nervous system, the incubation period of the virus in foxes is variable, but is generally taken to be around 28 days. Following this period the fox will be infectious for approximately 5 days, after which it will die [23].

Most of what is known about CDV infection comes from studies of domesticated dogs rather than by direct observation of disease in wild red foxes [7], [48]. CDV transmission between dogs is primarily through aerosolised respiratory excretions and it is likely that a similar route is followed in foxes [5], [7], [48]. The incubation period is variable, with these reports suggesting there is a wide interval in dogs of between 1 to 4 weeks, though it is likely to be around 7–10 days for most healthy individuals. Data from Arctic foxes (*Alopex lagopus*) suggest an incubation period of up to10 days [49]. Following the post-infection incubation period dogs are usually infective for a further 10 days, with the immune response that the host is able to mount being critical to the outcome of CDV infection. There is evidence that some infected individuals can shed virus for extended periods [5], [7] but whether this is true for foxes is not known. Whilst infection with rabies virus is almost invariably fatal for a fox, infection with CDV does not always result in death of canids [5], [50], and overall, the experimental mortality rate was found to be roughly 43%. Immunological evidence suggests that it is the capacity and robustness of the host immune response to the pathogen that determines the outcome of the infection [7], [51]. If they can mount a robust response then dogs can recover from CDV and maintain lifelong immunity to re-infection [5], [50]. If not, they die of the disease. Few up-to-date data are available concerning distemper mortality rates in wild red foxes. Kennedy [50], describing an outbreak of canine distemper in foxes raised in a ranch reported an 80% mortality rate amongst reared foxes with no indications of the fox species. Kelly and Sleeman [52] quoted a study in gray foxes (*Urocyon cinereoargenteus*) carried out in the south-eastern United States indicating that the most common cause of mortality was CDV, despite no specific data on mortality rates being presented. Other workers have found little evidence of CDV in fox populations [52]. If we take data relating to dogs as a guide [5], [7], [48] and supposing that wild red foxes may be slightly more immune-compromised than laboratory dogs then it is plausible to assume that roughly half of all CDV infections in foxes will result in host death. The remainder recover to a CDV-immune status and will eventually die of other causes.

#### Description of the model

Mathematical modelling of the rabies spread in fox populations [23]–[27] and PDV infection in seals [53] has demonstrated that useful epidemiological insight can be gained into the factors that influence empirically observed patterns of disease dynamics. Given the coarse nature of the data, we use non-spatial compartmental model as the basis for investigating the epidemiology and control of CDV and rabies in foxes. For the same reason, we also model *separately* each disease (but see Text S1 for a co-circulation model).

**Figure 3:**
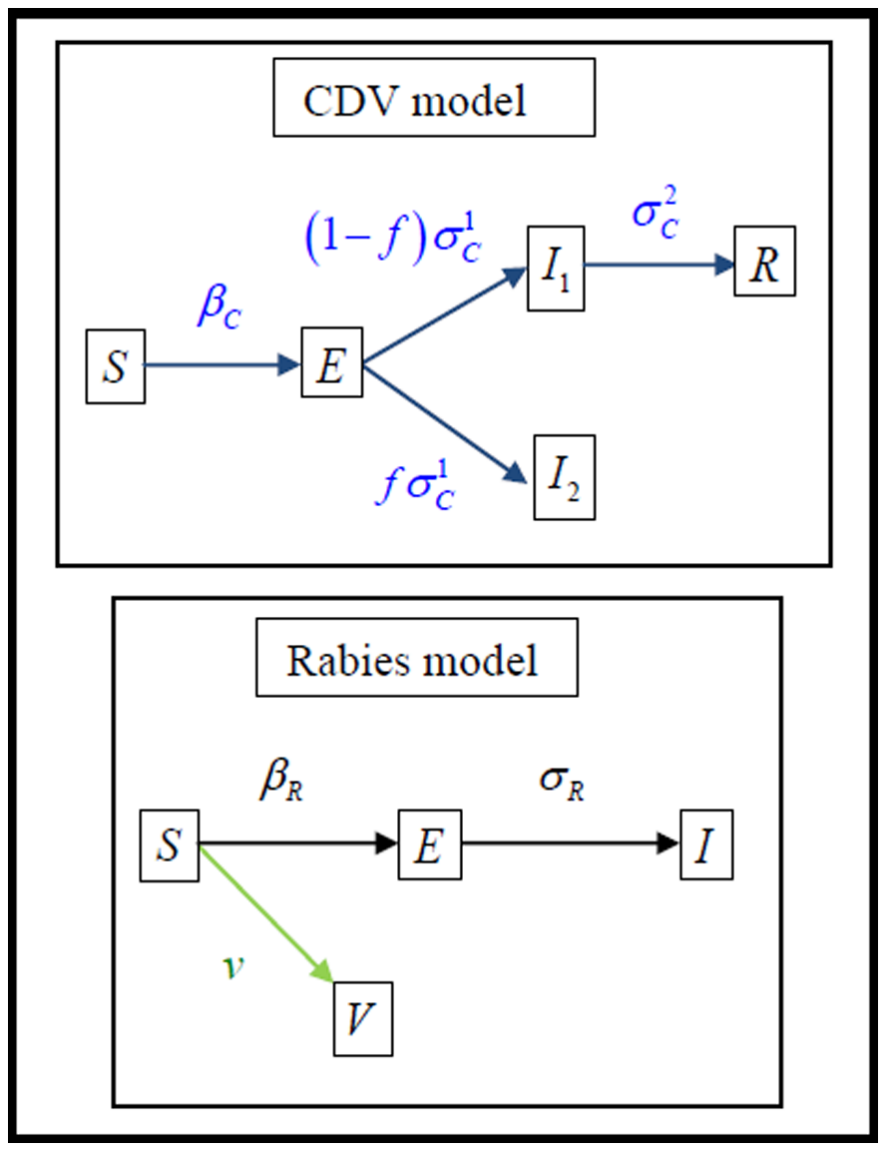
Flow chart between different compartments for the CDV and rabies models. By convention, each compartment is characterised by the density of susceptible 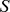, exposed 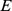, infectious 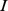 (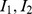 for CDV see methods) and recovered 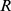 from CDV or vaccinated 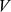 against rabies. In the flow chart, demography and virulence induced mortality are omitted leaving only the links between compartments due disease acquisition and development. The corresponding system of differential equations is presented in the materials and methods section.

First, concerning CDV, individuals can either be susceptible (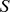), become exposed (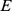) at rate 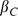, infectious (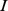) at rate 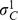 or recovered (*R*). A proportion 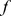 of CDV infectious foxes suffers from a strong form of CDV with added mortality 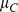 and will not recover, those are noted 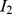. The other infectious foxes suffer a mild infection (without virulence) from which they can recover at rate 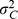, those are noted 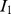. The corresponding system of differential equations is presented in Table 2 (see Figure 3 for a flow chart).

**Table 2:** Systems of differential equations describing the dynamics of CDV (top) and rabies (bottom).

CDV model						
		demography	Enter compartment	Leave compartment	Virulence induced mortality	
Susceptible	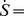	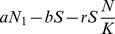		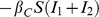		
Exposed	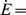	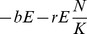	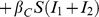	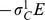		
Infectious	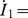	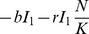	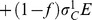	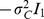		
	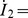	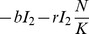	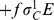		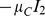	
recovered	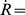	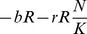	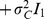			
With 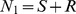						

By convention we note 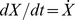. For ease of visualisation, in each equation, we separate the dynamics that were affected by either demographic, disease (entering or leaving a compartment due to disease development and virulence induced mortality) or vaccination factors. While the transmission parameter will be estimated, other parameters values can be found in Table 1.

Concerning rabies, following Anderson et al. 1981 [23], foxes can be either susceptible (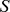), become exposed (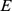) at rate 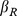 or infectious (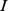) at rate 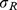. Infectious foxes do not recover and suffer an added mortality 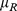. Because a strong vaccination programme was initiated in the winter 2009/2010, susceptible foxes may become vaccinated against rabies (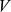) at rate 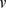. Again, Figure 3 presents a flow chart and Table 2 presents the corresponding system of equations:

#### Statistical inference of transmission parameters

Given a set of parameters, we can numerically solve each system of equations allowing us to determine the proportion of individual in each compartment as a function of time. When modelling CDV, the system was initially disease free and then a CDV epidemic started (at time 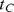). When modelling rabies, a disease free system was followed by a rabies epidemic (at 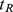) and then an oral rabies vaccination campaign (at 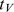).

Significant proportions of foxes sampled for either CDV or rabies were found dead and we note those 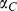 and 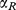. For the purpose of inference those proportions were calculated on a quarterly basis. Accounting for this, we attempt to find the transmission parameters (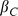 and 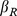) that best represented the data using a maximum likelihood approach.

Let us consider CDV modelling first. Initially we derived from our model the proportion of living foxes that would be expected to be either disease-free 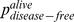 or positive for CDV 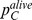. The RT-PCR (with direct immune-fluorescence assay) tests used to assess prevalence allowed us to detect foxes that were either exposed or infectious. We then derived the proportion of dead foxes 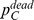 expected to test positive. For instance, the density of CDV positive dead foxes in the interval (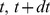) is:
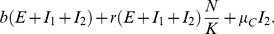



We divided the 5-year sampling period into 20 quarters. For each quarter, knowing the proportion of animals collected dead 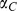, integrating the quantities above gives us the probability
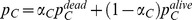
that a fox found in the field during this quarter was positive for CDV. Then, again for each quarter, we compared the observed number of positive (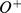) to an expected number of positive (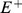), given the number sampled (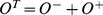) and the probability 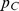 above. The likelihood of observing the data for a given set of parameters was calculated assuming that 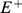 follows a binomial distribution with parameters 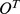 and 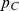.

We estimated the transmission parameters 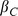 (as well as the onset of epidemic 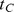) by maximising the likelihood. Values for other parameters were taken from Table 1.

A likelihood for the data on rabies was calculated using the same methods and used to estimate the transmission parameters 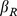 (as well as the onset of epidemics 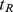). Values for other parameters were taken from Table 1.

Before and after the last quarter of 2009, the sampling clearly changed from passive to active surveillance. While data from active surveillance are more likely to be representative of the true incidence due to systematic protocols applied evenly across regions, data from passive surveillance are likely to be biased. Specifically there were indications that prior to the last quarter of 2009, reporting was likely biased toward reporting more CDV positive individuals. Given a probability ‘
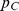
’ of CDV positive in the sampled population, we assumed that surveillance previous to the last quarter of 2009 was ‘*z*’ times more likely to collect a CDV positive compared to a negative fox. We thus modified ‘
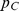
’ to account for this and obtain a new expected probability
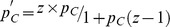
that a sample would be positive. The parameter ‘z’ was also estimated using the maximum likelihood approach described above.

Finally at the end of the 1^st^ quarter 2010, we also assumed an oral vaccination campaign was started (i.e. end of the 1^st^ vaccination campaign, with a 2^nd^ more comprehensive campaign started at the beginning of the 2^nd^ quarter, see above) and obtain a maximum likelihood estimate of the rate, *v*, of vaccination.

Once estimates were calculated, we performed non-parametric bootstraps to obtain 95% confidence intervals. During this procedure, the sampling distribution of the observed data is used to calculate new parameters' estimates, and 2.5 and 97.5% percentiles are calculated to obtain 95% confidence intervals. We finally present in Text S2 sensitivity analyses of our estimates to changes in the density of foxes in the population (*K*).

#### Implications for disease dynamics

Once the model was fitted, we could compare predicted prevalences of both diseases among dead and living foxes and compare these to observations. Furthermore, we were able to infer crudely the impact of the vaccination campaign by extrapolating from our model the expected prevalence of rabies had if the vaccination not occurred. Those values could be compared to prevalence observed and prevalence expected from the model including the vaccination.

Finally, following [23], the models presented above allowed us to determine for each disease the basic reproduction number, R_0_. For rabies the R_0_ can be calculated using:
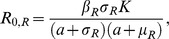
while for CDV the R_0_ can calculated using:
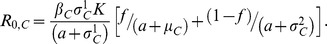



Finally this allowed us to determine, again for each disease, a critical fox density below which no epidemic would occur. For rabies, this critical density was:
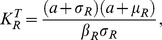
while for CDV it was:
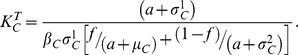



From this, we could infer that vaccinating a proportion 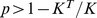 of the total population, would be sufficient to prevent the long term spread of the disease.

## Results

### CDV in wildlife in north-eastern Italy

The first notifications of CDV in the current outbreak in northern Italy occurred in May and August 2006 respectively in Friuli Venezia Giulia (Carnia, province of Udine) and in Alto Adige (Val Pusteria, province of Bolzano). In April 2007 the disease was confirmed in Veneto (Comelico Superiore, province of Belluno) and Trento province (Predazzo, province of Trento). In the 10 months following the initial detection, the outbreak involved four provinces and 21 municipalities of three different Italian regions [1]. Figure 1 shows the extent of the spread of CDV in wildlife in north-eastern Italy in the time interval since it was first reported through 2010.

The geographic spread of cases appears to be following a broadly south-westerly direction since the first cases in 2006, with the more recent cases (up to 2010) confirmed in Lombardia and in the proximity of the border with Lombardia. Cases were mainly localized in relatively isolated and wild areas of pre-Alpine and Alpine regions, but a few cases were also detected in urbanized areas of the region posing a potential risk to susceptible domestic pets.

**Figure 4:**
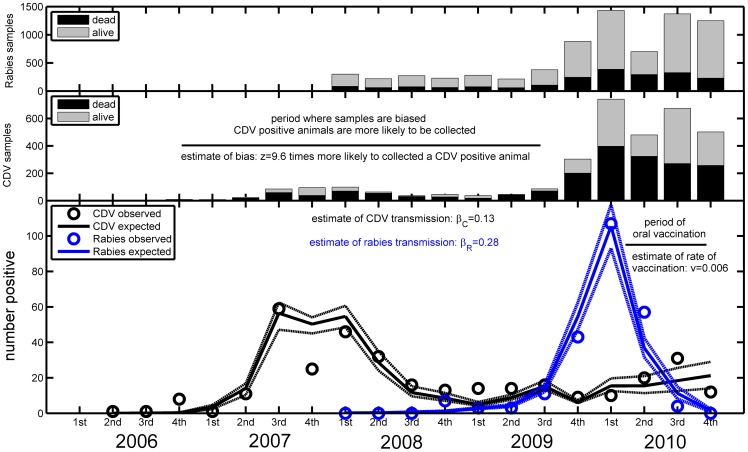
Observed (circle) and expected (solid line) positive cases of CDV (black) and rabies (blue) in the fox population from 2006 to 2010. The fit was obtained by maximum likelihood and corresponds to 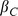 and 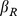 equal to 0.13 and 0.28 respectively. Both diseases were estimated to have started during the same quarter as they were first observed. The rate of vaccination was estimated at *v* = 0.006 and CDV samples prior to the last quarter of 2009 were estimated to be biased toward reporting *z* = 9.6 times more CDV positive foxes than CDV negative foxes. Based on each set of parameters' estimates from the bootstrap procedure we obtain each expected trajectory and thus obtain the 95% confidence interval around our model fit for each quarter, broken lines (this ignores temporal correlations within each trajectory). In the upper panels, for each disease, we present the sampling sizes for each quarter as well as the proportion of samples taken on either dead or living animals.

Up to December 2010, the presence of the CDV was confirmed in north-eastern Italy through laboratory diagnosis in 319 (10.7%) animals out of a total of 2967 samples collected. Just over 82% (82.1%, 262/319) of positive samples were collected from red foxes (*Vulpes vulpes*), followed by badgers (*Meles meles*) (16.3%, 52/319) and martens (*Martes foina*) (1.6%, 5/319). Considering foxes alone, during the whole sampling period (from 2006 to 2010) 55% of the samples were taken from animals found dead. Dividing the sampling period into quarters of a year, this proportion varied temporally from 0% to 100% (see Figure 4).

Extensive molecular evolutionary analysis of 96 Italian CDV positive samples collected from 2006 up to 2009 in Veneto, Friuli Venezia Giulia and Trentino Alto Adige regions yielded data on evolutionary dynamics of the Alpine wildlife CDV epidemic and revealed the emergence and spread of a novel CDV genetic group [1]. This new genetic group is likely to be widespread in Europe as demonstrated by phylogenetic analyses of all Italian CDV isolates (66 red foxes, 29 badgers (*Meles meles*) and 1 stone marten (*Martes foina*)) clustering with sequences obtained from Bavarian (South Germany) wild carnivores [13].

In northern Italy it is still unclear whether the introduction of CDV can be linked with the movement of domestic carnivore(s) as introduction of infected animals from neighbouring countries (i.e. Austria and Switzerland) through wildlife migration is possible. Benetka et al. [14] report positive cases of CDV in badgers and stone martens in Austria. Origgi et al. [15] describe virulent CDV strains detected in wildlife and a domestic dog in Switzerland that all shared a pathology somewhat distinct from the classical CDV.

### Rabies in wildlife in north-eastern Italy

As in previous fox rabies epidemics, the outbreak described herein has been linked to the epidemiological situation in the Balkan region. Re-introduction of rabies in Italy was thus a consequence of the westward spread of the infection front, as has been confirmed by phylogenetic analysis of the first Italian 2008 virus. The molecular analysis confirmed that the virus responsible for the rabies re-introduction in late 2008 belongs to the Western European group and is closely related to rabies virus samples collected from foxes in eastern neighbouring countries (Slovenia, Bosnia and Herzegovina and the former Federal Republic of Yugoslavia) [4].

Since its re-introduction in 2008, the fox rabies epidemic in north-eastern Italy has spread westwards (Figure 2). In November 2009 the epidemic reached the province of Belluno (Veneto region) which has been the most affected area with a total of 215 detected animal rabies cases, out of which 82% (176/215) were confirmed in foxes, as of 31/12/2010. Among foxes, 25% of all samples were collected on animals found dead and quarterly (of a year) variation ranged from 18 to 42% (Figure 4). Very few cases were detected more westerly than Belluno province (i.e. in Trento and Bolzano provinces) in 2010 and this is presumably the result of the oral fox vaccination campaigns undertaken in 2009 and 2010. Infection has predominantly affected the red fox population with few cases in other wildlife species, and occasional spill-over to domestic animals (See Table 3). No human rabies cases have been reported to date. In Italy no more cases have been reported since February 2011.

**Table 3:** Cases of rabies in domestic and wildlife animals in Italy, 2008–2010.

Animal species	2008	2009	2010
Red fox	8	61	172
Badger	1	2	12
Roe deer		1	8
Deer			1
Mustelid			4
Rodent			1
Cat			9
Dog		3	
Bovine			1
Donkey		1	
Horse			1
**Total**	**9**	**68**	**209**

Although many species were affected, including domestic animals, the main reservoir for rabies appears to be foxes.

At present, Austria and Switzerland are declared rabies free. Slovenia has not reported any cases of rabies in 2011 while Croatia reported 207 cases of rabies in wildlife population in the same year [21]. These data, and the evidence of introduction of the virus from Eastern Europe [3], [4], [54], sustain, with a reasonable certainty, the claim that no introduction of rabies infectious animals from northern countries (Austria and Switzerland) occurred from 2008 to date and re-enforce the evidence of a westwards spread of the outbreak in Italy since its first detection in 2008 in Friuli Venezia Giulia. Furthermore, the Alps cover all regions of north Italy and so represent a natural barrier to hinder and slow the spread of those diseases for which diffusion is associated with wildlife movement.

In north-eastern Italy, several oral fox vaccination campaigns using aerial distribution of vaccine baits have been implemented since 2009. This activity confirmed vaccination coverage, in terms of percentage of foxes that achieved protective antibody titres, ranging from 69% to 77% of the sampled animals. The timing, intensity and geographical coverage of the oral fox vaccination campaigns are extensively described in [42], [43], [55].

### Model results

Using mathematical models we are able to estimate transmission parameters for both diseases, quantify a bias in reporting CDV prior to 2010 and estimate the rate of vaccination for rabies achieved through the oral vaccination campaign. We develop a quantitative picture of the epidemiological situation and address questions of prevalence and disease elimination. Inference was based on *two* compartmental models and we model both diseases *separately*. In Text S1, we present a prototype full model that would allow co-circulation and co-infection of both diseases, allowing them to interfere with each other. However given the coarse nature of available data, we felt that the co-circulation model, despite showing encouraging results, proved to be too sensitive to the lack of data on the ecology of the fox population in north-eastern Italy and therefore the added complexity of this model was not currently justified. This model is thus not described in the main text but in Text S1.

#### Results from statistical inference

Figure 4 shows reasonable agreement between the expected and observed number of CDV and rabies positive foxes reported accounting for the proportion of samples taken on dead animals for each quarter of a year.

Using our maximum likelihood procedure, we estimate the rate of transmission of CDV, 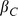, at 0.133 (95% CI: 0.129, 0.136) km^2^ per individual per day. This rate of transmission represents a compound parameter that depends on the rate of contact between individuals and the probability that such a contact between a susceptible and an infectious fox leads to a new infection. Our estimate suggests that such a contact leading to a new infection occurs approximately once a week. We also assessed that, prior to the last quarter of 2009, CDV samples were heavily biased toward reporting more CDV positive foxes. We quantified this bias and found that positive animals were 9.6 (95% CI: 8.1, 11.7) times more likely to be reported than CDV negative foxes.

Concerning rabies, our maximum likelihood estimate of the rate of transmission, 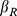, was 0.276 (95% CI: 0.272, 0.278) km^2^ per individual per day. Our estimate suggests that initially a rabid fox is in effective contact with susceptible foxes at the observed density on average approximately once every three days. We also estimated that fox vaccine baits intake, *v*, was 0.0057 (95% CI: 0.0048, 0.0067) per day. This allowed us to estimate that once the proportion of vaccinated foxes reaches a stable state (i.e. at equilibrium) 68% of the foxes would be immunised against rabies. This was in broad agreement with field observations which indicate that the vaccination campaign achieved immunisation of around 70% of foxes [41]–[43].

Finally, we found that while the estimate of the bias in early CDV samples and the estimate of the rate of vaccination were relatively insensitive to changes in fox density, both estimates of CDV and rabies transmission were negatively correlated to the density of fox assumed in the model. Full results of the sensitivity analyses to changes in the assumed density of foxes are presented in Text S2, together with a detailed justification of the value assumed for the fox density in the work presented.

#### Implications for disease dynamics

Using the estimates of our model, we could predict that the differences in prevalence expected among dead and living animals was very important, and this justified a posteriori the modelling work undertaken to account for the method of surveillance. For instance, our model estimated that among dead individuals the prevalence of CDV was expected to reach 29% during late 2007/early 2008 while expected to be less than 4% within living foxes. We also estimated that over the whole study period 15% of dead foxes would be positive compared to 2% among living foxes. This was in reasonable agreement with field observations which detected a prevalence of 10% among dead foxes compared to 4% among living foxes.

Similarly, the prevalence of rabies among dead foxes was expected to reach 11% during the first half of 2010, while being under 1% within living foxes. Also, we estimated that, between 2008 and 2010, 10% and 1% of foxes would be positive among dead and living animals respectively. This was in reasonable agreement with field observation which found 10% of dead foxes being positive compared to 4% of living foxes being positive.

Given our model, we were also able to extrapolate the influence of the vaccination program. Considering our estimate of *v* = 0.006 and of rabies transmission of 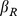 = 0.28, we were able to extrapolate the impact of the vaccination strategy. As expected, without vaccination the rabies epidemics would have been more dramatic. For instance during the last 3 quarters of 2010, setting *v* = 0 and keeping other parameters unchanged, we would have expected 15%, 10% and 8% of collected foxes to be rabid respectively in late spring, summer and autumn. As a comparison, only 8%, 0.3% and 0% were observed to be rabid during the respective time periods and our model predicted that 5.3%, 0.8% and 0.1% would be expected to be rabid when accounting for vaccination.

Finally from a theoretical stand point, and as in [23], we calculated the basic reproduction number, R_0_, of CDV at 1.26 in the north-eastern Italian setting. The rabies R_0_ in the Italian setting was also found to be 1.26. Therefore, both diseases have the same transmission potential as quantified by R_0_ but rabies has a shorter generation time (see Table 1) so that its spread is much faster than that of CDV. Additionally, the model presented together with the estimates of transmission parameters allowed us to determine for each disease a critical fox density below which no epidemic would occur. This critical density, *K^T^* , was 0.791 for rabies and 0.792 for CDV. From this, we conclude that vaccinating approximately 21% of foxes against rabies, or (hypothetically) 21% of foxes against CDV, would be sufficient to prevent the long term spread of the disease.

## Discussion

Although an early detection of diseases such as rabies or CDV in fox populations is strictly related to a systematic passive surveillance on found dead or symptomatic animals, assessment of disease incidence from such data is challenging since for example, the proportion of specimens testing positive against rabies in dead/suspect animals may largely overestimate incidence in the fox population. In this paper, we have developed a quantitative framework to obtain estimates of incidence that are corrected for such a bias. Our analysis also provided insights on the dynamics of rabies and CDV epidemics in the Italian setting between 2006 and 2010.

Of key interest for a wide range of epidemiologists and ecologists, we present a method to translate prevalence of infection in dead animals into prevalence among living individuals. This allows inference to be made when a given proportion of observations is made on dead animals. This proportion of dead animal observation may be temporally variable, a likely situation when passive surveillance programs are in place. This method could be useful in a range of situations as assessing prevalence among dead individuals might be logistically easier than assessing prevalence in living ones (e.g. from live trapping) in many ecological settings. In addition to the logistical advantage, collection and inference based on dead individuals is potentially more powerful as the prevalence of infection among dead might be much greater than among living individuals.

Basic non-seasonal compartmental models for rabies and CDV appear to give results consistent with the epidemiological field data. Very generally, the rate of rabies transmission appears more than twice that of CDV in red foxes. We provide the first estimate of CDV transmission among foxes at 0.13 km^2^ per individual per day suggesting that contact between an infectious and a susceptible that leads to a new infection occurs approximately once every week. This transmission rate allows us to make the first estimate of the reproductive number: a R_0_ of 1.26 in the north-eastern Italian setting. Additionally we confirm the rabies transmission parameter was close to 0.3 as previously found [23]–[28], and estimate the rabies R_0_ in the Italian setting was also 1.26. The similarity between both diseases' R_0_ might appear in contradiction with our estimation of transmission rates; however this apparent inconsistence can be explained by the faster generation time of rabies (see Table 1) and we conclude that both diseases are equally transmissible, as quantified by R_0_, but rabies, given its shorter generation time, leads to faster epidemics.

Our data originated through the implementation of a specific research programme aimed at investigating the phylogenetic characteristics of the CDV circulating in north-eastern Italy and, since 2008, under the national surveillance strategy for rabies. It is clear that pre 2010, the CDV prevalence observed was biased: a CDV positive individual was estimated to be circa 10 times more likely to be tested and reported. First we stress that this bias did not impede our ability to estimate the probability of transmission of CDV as 70% of all CDV samples were taken in 2010, when sampling appeared unbiased. Considering the bias prior to 2010, the foxes submitted to the laboratory came from passive surveillance samples, it is thus possible that foxes or carcasses presenting symptoms were more likely collected. Additionally, since passive surveillance was mainly on a voluntary basis, samples might also have been clustered where disease was most prevalent due to increased interest and motivation of hunters and gamekeepers. Concerning the period after the end of 2009, after the first cases of rabies in Friuli and even more after the first cases in Belluno province, surveillance became mandatory, including for hunted foxes, and culling campaigns were performed for vaccination monitoring, thus probably making the sampling more even. This highlights the importance of assessing the performance of the surveillance activities, in terms of overall sensitivity of the activities and consistency. Confirming this, the recent phylogenetic analyses from the same geographical area suggests that this rabies strain might have circulated unnoticed in wildlife earlier than its official detection in north-eastern Italy [54]. Consequently, the observed spatio-temporal distributions of confirmed cases, even though able to clearly suggest the broad pattern of the epidemics, may not represent accurately the underlying spatio-temporal spread of the infections, thus justifying the ‘simple’ approach presented here.

While the CDV epidemic remains ongoing [39], the rabies epidemic appears to be under control using oral vaccines. To control a rabies epidemic, the OIE ( ‘Office International des Epizooties’ known as World Organisation for Animal Health) and WHO (World Health Organisation) guidelines suggest that a population level immunity above 70% is required [56]. In line with this requirement, the coverage of the oral fox vaccination campaigns implemented since 2009 in northern Italy was observed to be around 70% of the fox population. From our modelling work, we estimated a vaccination rate of 0.006 per day allowing us to estimate that approximately 68% of foxes would be immunised at equilibrium. Given our estimate of transmission, our models indicated that at least 1/5 of the fox population will need to be vaccinated to eliminate rabies. While similar findings were also reported in [26] for low fox density, our estimate was slightly lower than most estimates in the literature [23], [57], [58] and reflected the lower density of foxes in the Italian setting. This suggests that a lower vaccination coverage may still be able to control the rabies outbreak. However, considering the uncertainty in the fox ecology means this needs to be more thoroughly tested before any drop in vaccination coverage is implemented. In the Italian setting, the wide geographical coverage and high proportion of foxes immunised [42], [44] appears to have played an important role in the control of the rabies epidemic and this offers hope concerning the feasibility of elimination of fox rabies in the wild, and its benefit in term of wildlife conservation.

There are areas where both diseases were detected in the same provinces (i.e. Belluno province) during the same period. The fact that both diseases share the same host suggests that the alteration of the fox population structure due to the presence of one disease could have affected the dynamics of the other disease. In this type of ‘ecological interference’ [59], the removal of individuals from the susceptible pool after an acute infection can influence the pattern of another disease. While due to scarce ecological and epidemiological data, we acknowledged the difficulties of this task, in Text S1 we provided a prototype model that allows both co-circulation and co-infection within the same fox population. This model can be used for inference of all parameters as above and we presented a fit of this extended model in Text S1. We felt that the increase in model complexity was not justified given the similarity in the estimates produced and in the fit, and the uncertainty surrounding both epidemics. However, according to this co-circulation model, an endemic CDV situation (i.e. once CDV reached equilibrium in the population) would not reduce enough the density of foxes to prevent an epidemic of rabies. If uncontrolled, the rabies epidemics would quickly out-compete the CDV epidemics, due to its faster generation time (see above), by reducing the fox density below its critical value (see Text S1 but also Text S2). We believe that an ‘ecological interference’ between the two diseases in Italy is possible and to strengthen this hypothesis there are currently indications of an increase in 2012 (therefore in absence of rabies outbreaks) of cases of CDV in foxes originating from north-eastern Italy [39]. Nevertheless, a proper assessment of such interference would need extensive field surveys to clarify the ecology of foxes, so that in the Italian context, the ecological interference, as defined in [59], of these two viral diseases on the fox population is still not clear.

The modelling work has highlighted those areas where empirical information is lacking if more refined models are to be developed and disease interventions are to be implemented. It is particularly relevant to consider and collect ecological data on foxes in the pre-Alpine and Alpine regions and specifically data on factors that affect the contact rate such as densities, home ranges, habitat use, movement and social organization of animals [28]. Also, topographic features such as rivers, lakes and mountains will have a significant effect on disease. There is clearly scope for more detailed studies, such as investigating the genotypic characteristics (presence of Simple Sequence Repeats or Short Tandem Repeats in the DNA) of the host population. These data could clarify fox movement patterns in the area in question and could be exploited in more detailed models. Finally our work highlighted the importance of compiling relevant information about the monitoring, i.e. the sampling effort, information on potential bias and the source of data (dead or living animals).

An immediate priority is establishing a better ecological picture of the fox population in pre-Alpine and Alpine areas of Italy and to gain more data and knowledge on the CDV epidemiology in wildlife carnivores. The modelling work has highlighted the importance of these data and they would be of considerable use in the future development of models of these epizootics. This is particularly important as the wide distribution of the novel CDV group, combined with the identification of a specific amino acid mutation which is believed to increase the ability of the virus to replicate in a wider host range [1], highlights the possible implications that the spread of this new sub-clade may have in terms of animal health, wildlife population dynamics and conservation of endangered wildlife species.

Finally these epidemics under study have spread in the same regions and a number of different wildlife species were infected. The presence of multiple hosts potentially capable of interspecies disease transmission likely influenced, to some extent, the persistence and distribution of these viruses [1], [28], [60], [61]. This is a key area in the ecology and dynamic of an infectious disease and we acknowledged that the presence of numerous hosts should be one of a key area of future model development.

## Supporting Information

Text S1 **In this supporting text, we develop a model of co-circulation that allows co-infection within a single fox population and use it to estimate transmission parameters of both diseases.**
(DOCX)

Text S2 **In this supporting text, we perform sensitivity analyses of our estimates to changes in the density of foxes.**
(DOCX)
