# Supplementary material for: Rabies and Canine Distemper Virus Epidemics in the Red Fox Population of Northern Italy (2006–2010)
Source: PLoS One. 2013 Apr 22;8(4):e61588. doi: 10.1371/journal.pone.0061588 (PMC3632604; doi:10.1371/journal.pone.0061588)
Supplement: Text S1 — In this supporting text, we develop a model of co-circulation that allows co-infection within a single fox population and use it to estimate transmission parameters of both diseases. (DOCX) [file pone.0061588.s001.docx]

**Text S1**

In this appendix, we develop a model of co-circulation that allows co-infection within a single fox population and use it to estimate transmission parameters of both diseases. This uses rabies and CDV epidemiological data gathered in the period 2006-2010; samples and data from 2011 and 2012 are currently being processed and analysed. In this model, the fact that both diseases share the same host implies that the alteration of the fox population structure due to the presence of one disease could have affected the dynamics of the other disease.

Description of the model

The epidemiological picture, involving the co-circulation of two viruses in the same wildlife host, is complex. Given the coarse nature of the data, we use non-spatial compartmental model as the basis for investigating the epidemiology and control of CDV and rabies in a single population of foxes.

As before, concerning CDV, individuals can either be susceptible ($S_{C}$subscript ‘C’ for CDV), become exposed ($E_{C}$) at rate $\beta_{C}$, infectious ($I_{C}$) at rate $\sigma_{C}^{1}$ or recovered ($R_{C}$). A proportion $f$ of CDV infectious foxes suffers from a strong form of CDV with added mortality $\mu_{C}$ and will not recover, those are noted $I_{C}^{2}$. The other infectious foxes suffer a mild infection (without virulence) from which they can recover at rate $\sigma_{C}^{2}$, those are noted $I_{C}^{1}$.

Concerning rabies, following Anderson et al. 1981 [23], foxes can be either susceptible ($S_{R}$, subscript ‘R’ for rabies), become exposed ($E_{R}$) at rate $\beta_{R}$ or infectious ($I_{R}$) at rate $\sigma_{R}$. Infectious foxes do not recover and suffer an added mortality $\mu_{R}$. Because a vaccination programme was initiated in the winter 2009/2010, susceptible foxes may become vaccinated against rabies ($V_{R}$) at rate $v$.

Figure S1: Flow chart between different compartments and corresponding system of differential equations (below). By convention, each compartment is characterised by the density of individual $N$ and its CDV and rabies status, i.e. the density of foxes susceptible for both disease is noted $N_{S_{C},S_{R}}$, the density of recovered from CDV and rabies infectious is noted: $N_{R_{C},I_{R}}$, etc. In the flow chart, demography and virulence induced mortality are omitted leaving only the links between compartments due disease acquisition and development. Black arrows and corresponding parameters relate to rabies, blue arrows and parameters relate to CDV, and green arrows and parameter relate to the influence of vaccination. The corresponding system of differential equations is presented and by convention we note . For ease of visualisation, in each equation, we separated the dynamics that were affected by either demographic, disease (entering or leaving a compartment due to disease development and virulence induced mortality) and vaccination factors.

The complete system of differential equation is:

with $N_{1}=N_{S_{C},S_{R}}+N_{R_{C},S_{R}}+N_{S_{C},V_{R}}+N_{R_{C},V_{R}}$.

Foxes can thus be classified into 20 different classes or compartments depending on their respective CDV and rabies status (see Figure S1 for a flow chart and the system of differential equations above). Our model allows co-circulation and co-infection of the diseases (see Box A1 for detailed description and a flow chart) and we assume that the fox status and course of infection for each disease was independent. The full model depends on 12 parameters, 3 for demography, 3 for rabies epidemiology, 5 for CDV and 1 for vaccination and while transmission parameters will be estimated as well as vaccination rate and a parameter accounting for bias in reporting CDV prior to the last quarter of 2009, other parameters values can be found in Table 3 (main text).

Statistical inference of transmission parameters

Given a set of parameters, we numerically solve the system of 20 equations allowing us to determine the proportion of individual in each compartment as a function of time. The system was initially disease free, then a CDV epidemic started (at time $t_{C}$), followed by a rabies epidemic (at $t_{R}$) and finally an oral rabies vaccination campaign (at $t_{V}$).

Statistical inference was undertaken as in the main text. First among living foxes, we derived the proportions (for each quarter) that were expected to be diseases free, positive either for CDV, for rabies or for both diseases. We then derived the corresponding proportions among dead individuals. This allowed the calculation of expected number of positive samples, $E^{+}$, that could be used in conjunction with the number of observed positive samples, $O^{+}$, and the total number of sample taken, $O^{T}=O^{-}+O^{+}$, to obtain a likelihood. Again the likelihood was based on an underlying binomial distribution.

We then estimate simultaneously both transmission parameters $\beta_{C}$ and $\beta_{R}$ (as well as the onset of both epidemics $t_{C}$ and$t_{R}$), the rate of vaccination, *v*, and the bias, *z*, in the collection of CDV prior to the last quarter of 2009 by maximising the likelihood. Values for other parameters were taken from Table 3.

Results from statistical inference

Using our maximum likelihood procedure, we estimate $\beta_{C}$ and $\beta_{R}$ respectively at 0.144 and 0.332 km^2^ per individual per day, with non-parametric bootstrap confidence intervals ranging respectively from 0.142 to 0.146 for CDV and from 0.329 to 0.336 for rabies. These rates of transmission were only slightly higher than when considering each disease separately (see main text). In the co-circulating model the density of susceptible to one disease is decreased due to the virulence of the other disease. Therefore to obtain the same incidence as observed, transmission in the co-circulating model must be higher than when considering the diseases separately with no interaction (e.g. as in the main text), to compensate for the decreased density of susceptible. It was thus expected that the co-circulating model would estimate transmission slightly higher. Figure S2 shows reasonable agreement between the expected and observed number of CDV and rabies positive; however the fit is very similar to that of figure 3 of the main text.

Vaccination rate was estimated at *v*=0.0065 (95% CI: 0.0057 – 0.0078) per day and the bias in reporting CDV prior to the last quarter of 2009 estimated at *z*=14.9 (95% CI: 12.3 – 18.2). We conclude that the estimated parameters between the single co-circulating model and the 2 separate models were roughly equivalent.

Implications for disease dynamics

As explained in the main text, we expected the prevalence of infection to be greater among dead animals. According to the model presented and the estimation derived, the maximum probability that a dead fox would test positive for both diseases was expected to be below 0.25%. This was in agreement with no such observation made on the field in the Italian context.

Finally, it can be shown analytically that, to a good approximation, for rabies and CDV to co-circulate requires $\frac{\beta_{C}\mu_{R}}{\beta_{R}\mu_{C}}>1$. According to the estimations presented from this co-circulation model, we conclude that the two diseases are not able to co-circulate steadily and simultaneously within the same population. However, assuming that (from Tables 2)$\mu_{R}/\mu_{C}\approx2$, then a slight increase of CDV transmission to$\beta_{C}>0.5\beta_{R}=0.17$ would permit co-circulation. This was borne out by the numerical solution of the model equations. We note that according to Text S2 this transmission rate would be reached assuming a lower fox density.


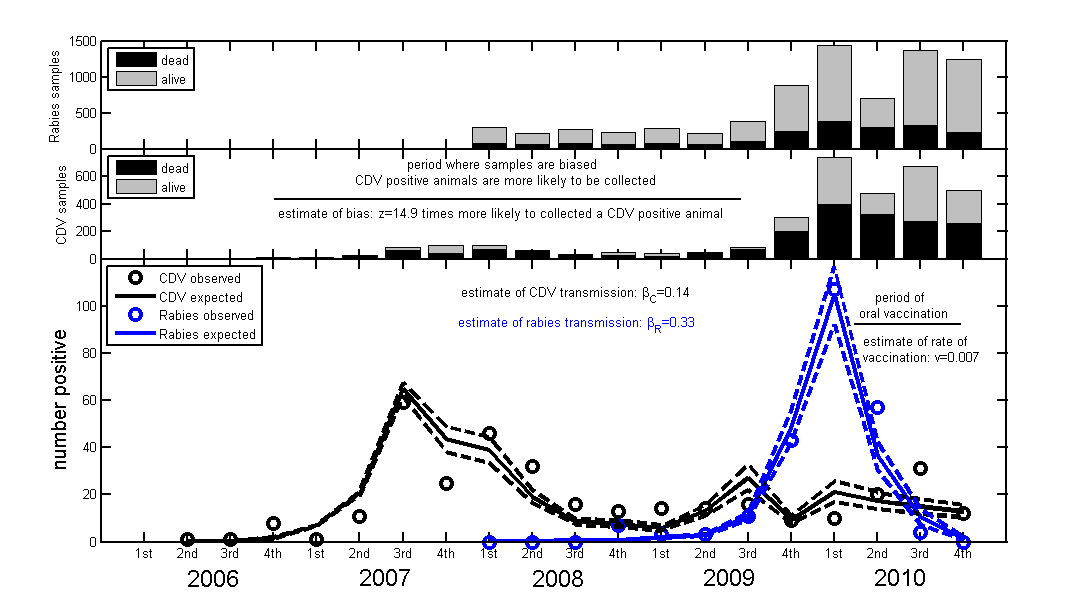


Figure S2: As in figure 3 of the main text, observed (circle) and expected (solid line) positive cases of CDV (black) and rabies (blue) in the fox population from 2006 to 2010. Both diseases were estimated to have started during the same quarter as they were first observed. In the upper panels, for each disease, we present the sampling sizes for each quarter as well as the proportion of samples taken on either dead or living animals.

In contrast to Figure 3, to obtain the fit presented, diseases were able to co-circulate and co-infect within the same population. This implies that the alteration of the fox population structure due to the presence of one disease would affect the dynamics of the other disease, a process termed ‘ecological interference’. The fit was obtained by maximum likelihood and corresponds to $\beta_{C}$ and $\beta_{R}$ equal to 0.14 and 0.33 respectively. The rate of vaccination was estimated at *v*=0.007 and CDV sample prior to last quarter of 2009 were estimated to be biased toward reporting *z*=14.9 times more CDV positive foxes than CDV negative foxes.
