# Supplementary material for: Rabies and Canine Distemper Virus Epidemics in the Red Fox Population of Northern Italy (2006–2010)
Source: PLoS One. 2013 Apr 22;8(4):e61588. doi: 10.1371/journal.pone.0061588 (PMC3632604; doi:10.1371/journal.pone.0061588)
Supplement: Text S2 — In this supporting text, we perform sensitivity analyses of our estimates to changes in the density of foxes. (DOCX) [file pone.0061588.s002.docx]

**Text S2**

In this appendix, we perform sensitivity analyses of our estimates to changes in the density of foxes. The estimates are as in the main text: transmissions parameters, bias in early CDV samples and vaccination rate. The full analyses of both the CDV and rabies models were performed as in the main text but assuming a fox density (*K*) of 0.5, 0.75, 1 (as in main text see Table 3), 1.25 and 1.5 foxes/km^2^.

|  | Sensitivity analysis on the CDV epidemic (CDV only model) | | | Sensitivity analysis on the rabies epidemic (rabies only model) | | |
| --- | --- | --- | --- | --- | --- | --- |
|  |  | estimate | 95% CI |  | estimate | 95% CI |
| *K*=0.5 | $\beta_{C}$ | 0.263 | 0.255 – 0.269 | $\beta_{R}$ | 0.544 | 0.5358 – 0.5480 |
|  | *z* | 10.6 | 8.9 – 13.0 | *v* | 0.0054 | 0.0038 – 0.0061 |
| *K*=0.75 | $\beta_{C}$ | 0.177 | 0.172 – 0.181 | $\beta_{R}$ | 0.365 | 0.3617 – 0.3673 |
|  | *z* | 9.9 | 8.4 – 12.2 | *v* | 0.0056 | 0.0047 – 0.0065 |
| *K*=1 | $\beta_{C}$ | 0.133 | 0.129 - 0.136 | $\beta_{R}$ | 0.275 | 0.272 to 0.278 |
|  | *z* | 9.6 | 8.1 - 11.7 | *v* | 0.0057 | 0.0048 to 0.0067 |
| *K*=1.25 | $\beta_{C}$ | 0.103 | 0.101 – 0.107 | $\beta_{R}$ | 0.222 | 0.2201 -.2237 |
|  | *z* | 15.5 | 9.8 – 17.5 | *v* | 0.0058 | 0.0050 – 0.0070 |
| *K*=1.5 | $\beta_{C}$ | 0.089 | 0.087 – 0.091 | $\beta_{R}$ | 0.184 | 0.1824 – 0.1874 |
|  | *z* | 9.0 | 7.8 – 11.0 | *v* | 0.0058 | 0.0051 – 0.0071 |

Table S1: Results of the sensitivity analyses. We performed the same analyses as in the main text on the 2 separate models of diseases dynamics while changing the assumed density of foxes.

Unsurprisingly, to predict the same incidence as observed while accounting for the fact that fox contacts increase at an increased density, our estimates for both the transmission of CDV, $\beta_{C}$, and rabies, $\beta_{R}$, decreases with increasing density. Based on estimates and their confidence interval, the bias in early CDV samples and the vaccination rate appear to be roughly independent of the fox density. One could argue that the fox density could have been estimated during the maximum likelihood procedure, however the data were insufficient and problems of convergence linked with local optima arose.

We still believe however that assuming a fox density of 1 fox/km^2^ was the best guess under the circumstance. As explained briefly in the methods, information on the spatial distribution of the fox population in northern Italy is limited. However data presented in Holmala and Kauhala [28] on the relationship between fox density and habitat type indicate that the “coniferous forest” habitat, the “field and forest” habitat and the “agricultural land” habitat are likely to support fox densities respectively of around 0.35 foxes/km^2^, 0.5 foxes/km^2^ and 1 foxes/km^2^. These habitats correspond broadly to the habitats where rabies and CDV cases were detected in Italy and would suggest a density slightly below 1. Unpublished data on the fox population density in the South Western area of Belluno province report an average density of 1.34 foxes/km^2^ [45]. These data are similar to data collected in Friuli Venezia Giulia and in Piacenza province by Meriggi and Rosa in 1991 [46]. However as any study of foxes is likely to be conducted where fox density is high, the true density over the whole area presented in this study is also likely to be lower. Therefore, it is likely that this red foxes population exist at a disease-free density of approximately 1 fox/km^2^.
